# Simplified versus traditional citrate anticoagulation in hemodialysis: Impact on clinical efficacy and patient prognosis, and correlative analysis

**DOI:** 10.1097/MD.0000000000047926

**Published:** 2026-03-20

**Authors:** Yunxia Wang, Jiajia Yang, Chun Wu

**Affiliations:** a People’s Hospital of Jinhu County, Huaian City, Jiangsu Province, China.

**Keywords:** clinical efficacy, correlative analysis, hemodialysis, prognosis, simplified citrate anticoagulation

## Abstract

Hemodialysis (HD) is a critical therapy for patients with end-stage renal disease, and the choice of anticoagulation protocol is crucial for its success. The impact of simplified citrate anticoagulation that notably eliminates the need for calcium supplementation on clinical efficacy and patient prognosis in comparison to traditional citrate anticoagulation has not been comprehensively elucidated. This study aimed to assess the impact of different anticoagulation protocols on HD patients. A prospective cohort study was conducted involving 109 HD patients, comprising a traditional citrate anticoagulation group (n = 57) and a simplified citrate anticoagulation group (n = 52). Various clinical and prognostic parameters including coagulation, inflammatory markers, cardiovascular parameters, treatment response, and patient-reported outcomes were assessed. Correlative analyses were performed to explore the associations between different anticoagulation protocols and clinical and prognostic parameters. The simplified citrate anticoagulation group showed significantly prolonged activated partial thromboplastin time, higher levels of fibrinogen, interleukin-6, and white blood cell count compared to the traditional citrate anticoagulation group. The simplified citrate anticoagulation group also had a higher left ventricular ejection fraction, a higher rate of partial response (61.54% vs 31.58%), and a lower rate of stable disease (32.69% vs 63.16%). Additionally, the quality of life score was higher in the simplified citrate anticoagulation group. Correlative analysis revealed significant associations between the anticoagulation protocols and various clinical and prognostic parameters in HD patients. The simplified citrate anticoagulation protocol for HD demonstrates advantages across various domains, including coagulation parameters, inflammatory markers, cardiovascular parameters, treatment response, and patient prognosis.

## 1. Introduction

End-stage renal disease (ESRD) represents a significant public health challenge, with a growing prevalence worldwide.^[1,2]^ Patients with ESRD often require hemodialysis (HD) as a life-sustaining treatment modality to manage the complications of renal failure.^[3]^ Hemodialysis involves the extracorporeal removal of waste products and excess fluids from the blood, and it is integral to the care of individuals with ESRD.^[4]^ Anticoagulation is essential during HD to prevent clotting within the extracorporeal circuit (ECC), ensuring the efficiency and safety of the procedure.^[5,6]^ In conventional HD practice, systemic anticoagulation with heparin (either unfractionated or low molecular weight) were remain the gold standard for maintaining circuit patency during maintenance HD sessions, as endorsed by the KDIGO (Kidney Disease Improving Global Outcomes) 2012 Clinical Practice Guidelines.^[7]^ However, approximately 15% to 20% of chronic HD patients present contraindications to heparin therapy, including active bleeding risks, heparin-induced thrombocytopenia, or perioperative status.^[8]^ This clinical reality has driven the adoption of regional citrate anticoagulation (RCA), which achieves localized anticoagulation through calcium chelation while minimizing systemic effects. Among the various anticoagulation strategies employed in HD, citrate-based anticoagulation has gained prominence due to its efficacy and safety profile. Citrate acts as an anticoagulant by chelating ionized calcium in the blood, thereby inhibiting the coagulation cascade within the HD circuit.^[9-11]^ The use of citrate as an anticoagulant offers several advantages, including reduced systemic anticoagulation, minimized risk of bleeding, and preservation of dialyzer filter patency.^[12-14]^ Traditional citrate anticoagulation protocols have been established and employed in clinical practice, providing effective clotting prevention during HD.^[15,16]^ However, these protocols often require meticulous monitoring and dose adjustments to ensure optimal anticoagulation while minimizing the risk of adverse events.

In recent years, there has been increasing interest in simplified citrate anticoagulation protocols, which aim to streamline the administration and monitoring of citrate during HD. The proposed simplified citrate anticoagulation (sRCA) regimen eliminates calcium-free dialysate requirements and reduces procedural steps from 8–12 to 3, while maintaining equivalent circuit survival rates (4.1 vs 4.3 hours, *P* = .62).^[17]^ Simplified protocols offer the potential for easier implementation, reduced complexity of monitoring, and improved resource utilization. However, the impact of simplified citrate anticoagulation on clinical efficacy and patient prognosis in comparison to traditional citrate anticoagulation has not been comprehensively elucidated. To address these knowledge gaps, we conducted a prospective cohort study to compare the clinical efficacy and patient prognosis of traditional citrate anticoagulation with those of simplified citrate anticoagulation in HD patients. This study aimed to assess the impact of these anticoagulation protocols on coagulation parameters, inflammatory markers, cardiovascular parameters, treatment response, and patient-reported outcomes. Additionally, we conducted correlative analyses to explore the associations between different anticoagulation protocols and various clinical and prognostic parameters in HD patients.

## 2. Materials and methods

### 2.1. Study design and participants

This study was approved by our Hospital Institutional Review Board and Ethics Committee and the protocol of this study follows the guidelines outlined in the Declaration of Helsinki and all subsequent amendments. All patients granted informed consent and volunteered to participate in this study. Patients who were scheduled for HD from June 2022 to June 2023 at our hospital were selected. Patients were grouped based on the different anticoagulation protocols they received, namely the traditional citrate anticoagulation group and the simplified citrate anticoagulation group. The choice of treatment involved several steps to ensure patient preferences were respected and considered. Initially, the physicians provided comprehensive information about the existing treatment options, including their benefits and potential risks, to all patients in a clear and understandable manner, ensuring that the patients fully understood their choices. Subsequently, the patients and physicians made decisions together, openly and honestly discussing the available treatment options, taking into account the patients’ medical conditions, personal values, and preferences. Importantly, all patient decisions were made within the framework of medical ethics, ensuring that patient autonomy and informed consent were effectively upheld throughout the decision-making process.

The following criteria must be met for patients to be eligible for inclusion in this study: Diagnosis of ESRD and stable patient condition; no abnormalities during HD treatment; age between 50 and 85 years; normal mental and cognitive function; complete medical records. The following criteria were employed to exclude patients with systemic and blood-borne infectious diseases, poor compliance with medical advice and premature withdrawal, severe liver dysfunction, irreversible hypotension, patients unable to cooperate, those with malignant tumors or severe cardiac or pulmonary abnormalities, and contraindications to local citrate anticoagulation.

### 2.2. Anticoagulation protocols

The simplified citrate anticoagulation protocol involves the following specific procedures: during HD, RCA is pumped into the artery end of the extracorporeal blood circuit (ECC) at a blood flow rate of 150 mL/min with a 4% citrate solution at a rate of 200 mL/h (1.33 times the blood flow rate). Correlation (Ca 1.25 mmol/L) is used for dialysis, and the citrate is discontinued 30 minutes before the end of treatment, with a blood flow rate adjusted to 220 mL/h for HD. In the traditional empirical-regional citrate anticoagulation group, citrate is empirically administered at a certain dosage (1.2–1.5 times the blood flow rate) at 180 mL/min, with a 4% citrate solution at a rate of 240 mL/h and calcium gluconate at 14.4 mL/h (6.1% of the citrate flow rate), followed by adjustments to the infusion rates based on monitoring the patient’s extracorporeal and intra-body ionized calcium levels.

### 2.3. Data collection

Patient data, including age, duration of dialysis, hemoglobin, serum albumin, body mass index, patient prognosis (hospitalization days, mortality rate, quality of life score, residual renal function [RRF], and cardiovascular event rate) were collected by the same experienced physician.

### 2.4. Blood indices

Three milliliters of fasting venous blood were drawn from the patients’ elbow vein within 30 minutes after HD session completion. After serum separation, an automated biochemical analyzer (BS-280, Mindray, Shenzhen, China) was used to analyze white blood cell count, hemoglobin, platelet count, serum albumin, blood calcium, blood phosphorus, blood potassium, lymphocyte count, d-dimer, fibrinogen, and brain natriuretic peptide levels. Levels of cardiac troponin I were measured using chemiluminescence immunoassay, and levels of inflammatory factors, including interleukin-6 (IL-6; ab178013, Abcam, Cambridge, UK), tumor necrosis factor-α (ab181421, Abcam), and C-reactive protein (CRP; ab260058, Abcam), were determined using enzyme-linked immunosorbent assay. Neutrophil count was assessed using an automated hematology analyzer (XE2100, SYSMEX, Kobe, Japan). Moreover, 1.8 mL fasting venous blood sample was obtained from the subjects, simultaneously using 0.2 mL of sodium citrate for anticoagulation. After shaking, the sample was then centrifuged at 3000 r/min for 10 minutes. The operational steps involved heating the reagent to 37°C, followed by placing the test specimen tube in a 37°C environment. Patient sample standard and quality control plasma, both 50 μL, were used. Prothrombin time was incubated with the reagent at 37°C for 60 seconds, with the reagent at 100 μL. Activated partial thromboplastin time (aPTT) was incubated with the reagent at 37°C for 180 seconds, with the reagent at 50 μL. Timing commenced after adding the reagent for comprehensive clotting time calculation. During the study period, routine clinical monitoring included visual inspection of the ECC for coagulation events. However, no standardized protocol for quantifying bloodline or dialyzer coagulation was implemented, and these parameters were not systematically recorded in the study database.

### 2.5. Doppler ultrasound assessment

The left ventricular ejection fraction (LVEF) was calculated using a full digital color Doppler ultrasound diagnostic instrument (Voluson E8, GE Healthcare Austria GmbH & Co. OG, Zipf, Austria) employing the biplane Simpson method.

### 2.6. Quality of life score

The 36-Item Short Form Health Survey,^[18]^ is a widely used tool for assessing health-related quality of life, was administered at post-intervention. This validated instrument evaluates 8 health domains: physical functioning, role limitations due to physical health, bodily pain, general health perceptions, vitality, social functioning, role limitations due to emotional problems, and mental health.^[18]^ Each question is scored on a 5-point scale ranging from “Excellent,” “Very Good,” “Good,” “Fair,” to “Poor.” Each question’s score ranges from 0 to 100, with higher scores indicating lesser impact of the related issue. The Cronbach α was 0.85.

### 2.7. Residual renal function assessment

Residual renal function was evaluated by measuring the glomerular filtration rate at the end of the study period. Glomerular filtration rate was determined using 24-hour urinary creatinine clearance, calculated from serum and urine creatinine concentrations quantified via enzymatic assays. To ensure accuracy, participants were instructed to adhere to standardized protocols for urine collection, and incomplete samples were excluded from analysis. Serum creatinine levels were measured using a calibrated automated analyzer, with results normalized to body surface area.

### 2.8. Criteria for inflammatory and cardiovascular treatment responses

The criteria for treatment responses were defined as follows: Complete response was determined by IL-6 levels below 5 pg/mL and CRP levels below 3 for inflammation, and an improvement of at least 5% in LVEF for cardiovascular status. Partial response (PR) was characterized by an increase in IL-6 levels of at least 30% for inflammation, and no new onset of arrhythmias for cardiovascular status. Stable disease (SD) was indicated by biomarker variations within ±20% for inflammation, and an LVEF change of <5% for cardiovascular status. Progressive disease was defined by an increase in IL-6 levels of at least 50% for inflammation, and new onset or worsening of heart failure for cardiovascular status.

### 2.9. Statistical analysis

Data analysis was performed using SPSS 29.0 statistical software (SPSS Inc, Chicago). Categorical data were presented in the form of (n [%]). For sample sizes ≥ 40 and theoretical frequencies (*T*) ≥ 5, the chi-square test was carried out using the basic formula. When the sample size was ≥40 and 1 ≤ *T* < 5, a corrected formula was applied for the chi-square test. In cases where the sample size was <40 or the theoretical frequency was *T* < 1, statistical analysis was conducted using Fisher exact test. The Shapiro–Wilk method was employed for testing the normal distribution of continuous variables. For normally distributed continuous variables, they were presented in the form of mean ± standard deviation, and a corrected variance *t* test was utilized. Non-normally distributed data were represented in the form of median (25th percentile, 75th percentile), and the Wilcoxon rank-sum test was used. The relationship between continuous variables such as serum calcium, aPTT, fibrinogen, IL-6, white blood cell count, LVEF, quality of life score, and the clinical efficacy and prognosis of HD patients was analyzed using Pearson correlation analysis. The relationship between categorical variables related to efficacy and the clinical efficacy and prognosis of HD patients was assessed using Spearman correlation analysis. A 2-tailed *P*-value < .05 was considered statistically significant.

## 3. Results

### 3.1. Baseline characteristics

The baseline characteristics of patients in the traditional citrate anticoagulation group (n = 57) and the simplified citrate anticoagulation group (n = 52) were comparable. There were no significant differences, and body mass index between the 2 groups (*P* > .05). In addition, the laboratory parameters before HD were observed no statistically significant differences between the traditional citrate anticoagulation group and simplified citrate anticoagulation group. Specifically, there were no significant variances in hemoglobin levels, serum phosphorus concentrations, serum potassium levels, and serum albumin concentrations between the 2 groups. These results indicate that the 2 groups were well-matched at baseline, providing a basis for comparative analysis of the clinical efficacy and patient prognosis between traditional and simplified citrate anticoagulation methods in HD patients (Table 1).

**Table 1:** Baseline characteristics of traditional citrate anticoagulation and simplified citrate anticoagulation groups.

Parameter	Traditional citrate anticoagulation (n = 57)	Simplified citrate anticoagulation (n = 52)	*t*	*P* value
Age (yr)	58.74 ± 6.21	58.52 ± 5.93	0.193	.847
Duration of dialysis (mo)	27.46 ± 3.78	26.91 ± 4.22	0.709	.480
Hemoglobin (g/dL)	11.82 ± 1.29	11.96 ± 1.42	0.517	.606
Serum albumin (g/L)	3.98 ± 0.42	4.05 ± 0.36	0.968	.335
Body mass index (kg/m^2^)	24.67 ± 2.31	24.14 ± 2.09	1.259	.211
Laboratory parameters before HD				
Hemoglobin (g/dL)	11.78 ± 0.91	11.93 ± 0.88	0.868	.388
Serum phosphorus (mg/dL)	4.12 ± 0.56	4.05 ± 0.51	0.708	.481
Serum potassium (mEq/L)	4.21 ± 0.45	4.15 ± 0.42	0.762	.448
Serum albumin (g/dL)	3.89 ± 0.21	3.95 ± 0.19	1.541	.126

HD = hemodialysis.

### 3.2. Coagulation parameters

The aPTT was significantly prolonged in the simplified citrate anticoagulation group compared to the traditional citrate anticoagulation group (*P* = .029). Additionally, there were significant differences in the levels of fibrinogen, which were higher in the simplified citrate anticoagulation group than in the traditional citrate anticoagulation group (*P* = .009). However, no significant variations were noted in the prothrombin time, platelet count, and d-dimer levels between the 2 groups (*P* > .05; Table 2). These findings suggest that simplified citrate anticoagulation may affect certain coagulation parameters differently compared to traditional citrate anticoagulation.

**Table 2:** Coagulation parameters of traditional citrate anticoagulation and simplified citrate anticoagulation groups.

Parameter	Traditional citrate anticoagulation (n = 57)	Simplified citrate anticoagulation (n = 52)	*t*	*P* value
Prothrombin time (s)	13.76 ± 1.89	13.42 ± 1.71	0.982	.328
Activated partial thromboplastin time (s)	28.83 ± 2.67	29.89 ± 2.34	2.207	.029
Platelet count (10^9^/L)	210.58 ± 25.67	208.94 ± 23.81	0.346	.730
Fibrinogen (g/L)	3.52 ± 0.48	3.75 ± 0.41	2.667	.009
d-dimer (mg/L)	0.40 ± 0.07	0.39 ± 0.06	0.524	.601

### 3.3. Inflammatory markers

Higher levels of IL-6 and white blood cell count (*P* < .05) were detected in the simplified citrate anticoagulation group compared to the traditional citrate anticoagulation group. However, no significant differences were evident in CRP, tumor necrosis factor-α, and neutrophil-to-lymphocyte ratio between the 2 groups (*P* > .05; Table 3). These findings suggest that the simplified citrate anticoagulation method may elicit differential effects on certain inflammatory markers, indicating the influence on the inflammatory response in HD patients.

**Table 3:** Inflammatory markers in traditional citrate anticoagulation and simplified citrate anticoagulation groups.

Parameter	Traditional citrate anticoagulation (n = 57)	Simplified citrate anticoagulation (n = 52)	*t*	*P* value
CRP (mg/L)	5.62 ± 0.87	5.39 ± 0.81	1.443	.152
IL-6 (pg/mL)	4.57 ± 0.63	4.91 ± 0.69	2.705	.008
TNF-α (pg/mL)	3.277 ± 0.42	2.142 ± 0.39	1.731	.086
White blood cell count (10^9^/L)	6.42 ± 0.92	6.89 ± 0.88	2.693	.008
Neutrophil-to-lymphocyte ratio	2.86 ± 0.37	2.78 ± 0.35	1.063	.290

CRP = C-reactive protein, IL-6 = interleukin-6, TNF-α = tumor necrosis factor-alpha.

### 3.4. Cardiovascular parameters

The LVEF was significantly higher in the simplified citrate anticoagulation group compared to the traditional citrate anticoagulation group (*P <* .05). However, no significant differences were noted in systolic and diastolic blood pressure, troponin-I level, and brain natriuretic peptide between the 2 groups (*P *> .05; Table 4). These findings suggest that the simplified citrate anticoagulation method may have a differential impact on certain cardiovascular parameters, particularly left ventricular function, in HD patients.

**Table 4:** Cardiovascular parameters in traditional citrate anticoagulation and simplified citrate anticoagulation groups.

Parameter	Traditional citrate anticoagulation (n = 57)	Simplified citrate anticoagulation (n = 52)	*t*	*P* value
Systolic blood pressure (mm Hg)	125.68 ± 8.32	126.41 ± 7.91	0.472	.638
Diastolic blood pressure (mm Hg)	72.34 ± 5.91	71.98 ± 5.67	0.320	.749
Left ventricular ejection fraction (%)	57.02 ± 4.61	58.94 ± 4.39	2.223	.028
Troponin-I level (ng/mL)	0.07 ± 0.03	0.06 ± 0.03	1.718	.089
Brain natriuretic peptide (pg/mL)	50.72 ± 7.91	49.86 ± 7.42	0.584	.561

### 3.5. Effectiveness analysis

**Figure 1.**
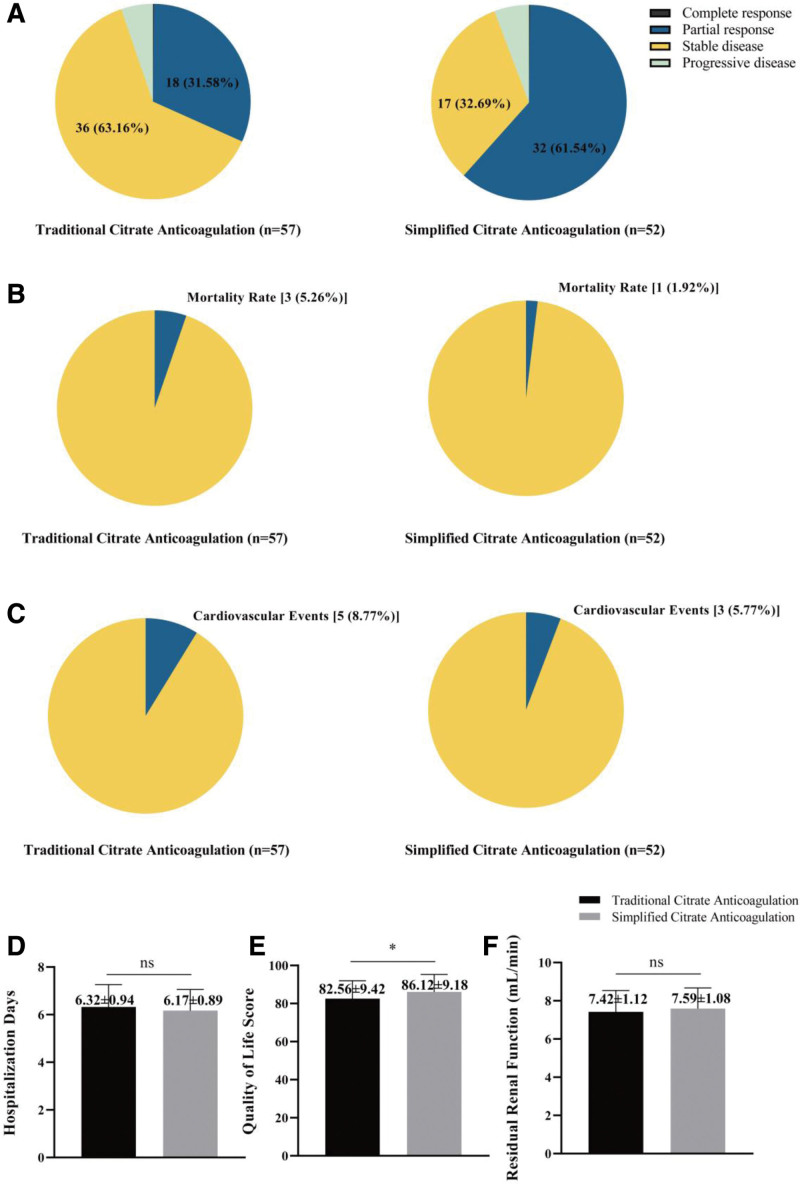
Effectiveness analysis of anticoagulation method on the clinical efficacy and patient prognosis. (A) Comparison of curative effect between traditional citrate anticoagulation and simplified citrate anticoagulation 2 groups. Black area means complete response; blue area means partial response; yellow area means stable disease; cyan area means progressive. (B) The mortality rate between traditional citrate anticoagulation and simplified citrate anticoagulation 2 groups. Blue area means the percentage of the mortality rate. (C) The cases of the cardiovascular events between traditional citrate anticoagulation and simplified citrate anticoagulation 2 groups. Blue area means the percentage of the cardiovascular events. (D–F) Hospitalization days, quality of life score and residual renal function between traditional citrate anticoagulation and simplified citrate anticoagulation 2 groups. *P < .05; ns means no significant differences between 2 groups, *P* > .05.

The simplified citrate anticoagulation group exhibited a higher rate of PR compared to the traditional citrate anticoagulation group (61.54% vs 31.58%), while the traditional citrate anticoagulation group demonstrated a higher rate of SD compared to the simplified citrate anticoagulation group (63.16% vs 32.69%). Both groups had a similar proportion of patients with progressive disease (Fig. 1A). These findings suggest that the simplified citrate anticoagulation method may be associated with a higher rate of PR and a lower rate of SD compared to traditional citrate anticoagulation in HD patients, emphasizing the impact of the anticoagulation method on the clinical efficacy and patient prognosis. Moreover, the simplified citrate anticoagulation group exhibited a higher quality of life score compared to the traditional citrate anticoagulation group (Fig. 1E). However, no significant differences were evident in hospitalization days, RRF between the 2 groups (Fig. 1D, F). Mortality rate and the incidence of cardiovascular events also had no significant differences between 2 groups (Fig. 1B, C). These results suggest that the simplified citrate anticoagulation method may contribute to an improved quality of life in HD patients when compared to traditional citrate anticoagulation.

### 3.6. Correlation analysis

The correlative analysis revealed significant associations between different anticoagulation protocols and various clinical and prognostic parameters in HD patients. Specifically, the simplified citrate anticoagulation protocol demonstrated a positive correlation with aPTT, fibrinogen levels, IL-6 levels, white blood cell count, and LVEF. Additionally, a negative correlation was observed between the simplified citrate anticoagulation protocol and curative effect, indicating a potential impact on treatment response. Furthermore, the simplified citrate anticoagulation protocol displayed a positive correlation with the quality of life score, highlighting its potential influence on patient-reported outcomes (Table 5). These findings underscore the interconnectedness of anticoagulation protocols with various clinical efficacy and patient prognosis indicators.

**Table 5:** Correlative analysis of different anticoagulation protocols with clinical efficacy and patient prognosis in HD.

Parameter	*r*	*R* ^2^	*P* value
Activated partial thromboplastin time (s)	0.207	0.043	.030
Fibrinogen (g/L)	0.248	0.061	.009
Interleukin-6 (pg/mL)	0.253	0.064	.008
White blood cell count (10^9^/L)	0.251	0.063	.008
Left ventricular ejection fraction (%)	0.21	0.044	.029
Curative effect	−0.248	0.062	.009
Quality of life score	0.189	0.036	.048

HD = hemodialysis.

## 4. Discussion

Hemodialysis is a key therapy for patients with ESRD, and the choice of anticoagulation protocol is a crucial factor in ensuring the success of this treatment modality.^[19-21]^ This study aimed to compare the impact of the simplified citrate anticoagulation method with the traditional citrate anticoagulation method on clinical efficacy and patient prognosis in HD patients. The study observed significant differences in coagulation parameters and inflammatory markers between the traditional and simplified citrate anticoagulation groups. Specifically, the simplified citrate anticoagulation group exhibited a significantly prolonged aPTT and higher levels of fibrinogen, IL-6, and white blood cell count compared to the traditional citrate anticoagulation group. These findings are consistent with the known effects of citrate anticoagulation on coagulation and inflammatory pathways.^[22,23]^ Citrate exerts its anticoagulant effect by chelating ionized calcium, thereby interrupting the coagulation cascade.^[24-26]^ The observed differences in coagulation parameters and inflammatory markers between the 2 anticoagulation protocols suggest that simplified citrate anticoagulation may elicit differential effects on coagulation and inflammatory pathways compared to traditional citrate anticoagulation.

The simplified citrate anticoagulation protocol streamlines the administration and monitoring of citrate during HD, making it potentially easier to implement and manage.^[27,28]^ This simplified approach could lead to better adherence to the prescribed anticoagulation regimen, resulting in improved coagulation control and reduced clotting within the HD circuit.^[29]^ Traditional citrate anticoagulation protocols often require meticulous monitoring and dose adjustments, leading to potential variability in anticoagulation efficacy.^[30]^ In contrast, the standardized and simplified nature of the new protocol may reduce variability and lead to more consistent anticoagulation levels, thereby contributing to improved clinical outcomes. The study also revealed significant differences in LVEF between the traditional and simplified citrate anticoagulation groups. Notably, the simplified citrate anticoagulation group exhibited a higher LVEF compared to the traditional citrate anticoagulation group. These findings underscore the potential impact of citrate anticoagulation protocols on cardiovascular parameters in HD patients. The improvement in LVEF in the simplified citrate anticoagulation group may indicate a beneficial effect on cardiac function, this was also consistent with the conclusions of previous literature,^[31,32]^ highlighting the importance of considering cardiovascular outcomes in the selection of anticoagulation protocols for HD.

The observed elevation of IL-6 in the simplified citrate anticoagulation group should be interpreted cautiously. This increase may represent a transient inflammatory response related to extracorporeal circulation or metabolic adjustments during dialysis rather than a sustained pathological inflammatory state. At present, there is insufficient longitudinal evidence to conclude that this finding indicates an adverse clinical signal. Future studies incorporating serial inflammatory monitoring are warranted to clarify its clinical relevance.

The clinical efficacy and patient prognosis differed between the traditional and simplified citrate anticoagulation groups. The simplified citrate anticoagulation group demonstrated a higher rate of PR and a lower rate of SD compared to the traditional citrate anticoagulation group. The simplified citrate anticoagulation method may effectively mitigate systemic anticoagulation effects, as evidenced by the reduction in bleeding risk and preserved dialyzer filter patency, there were also reported in relevant literature.^[33]^ This could lead to improved cardiovascular parameters, reduced inflammatory responses, and better treatment response due to decreased systemic impact on the patient. Additionally, the simplified citrate anticoagulation group exhibited a higher quality of life score compared to the traditional citrate anticoagulation group. These findings suggest that the simplified citrate anticoagulation protocol may be associated with improved treatment response and patient-reported outcomes in HD. The impact on patient-reported outcomes, as evidenced by the quality of life score, is particularly notable and emphasizes the importance of considering patient-reported outcomes in evaluating anticoagulation protocols for HD.

The correlative analysis highlighted several associations between the anticoagulation protocols and various clinical and prognostic parameters. Notably, the simplified citrate anticoagulation protocol demonstrated positive correlations with aPTT, fibrinogen levels, IL-6 levels, white blood cell count, and LVEF. Furthermore, a negative correlation was observed between the simplified citrate anticoagulation protocol and treatment response. These findings provide insight into the interconnectedness of anticoagulation protocols with clinical and prognostic indicators and emphasize the need for comprehensive assessment of these parameters in the context of HD treatment. The findings of this study have several clinical implications for the management of HD patients. The differential impact of traditional and simplified citrate anticoagulation protocols on coagulation, inflammatory markers, cardiovascular parameters, treatment response, and patient-reported outcomes underscores the importance of individualizing anticoagulation strategies in HD. Clinicians should consider the potential effects of anticoagulation protocols on clinical and prognostic parameters when making treatment decisions for HD patients. Moreover, future research should further explore the mechanisms underlying the observed differences between traditional and simplified citrate anticoagulation and investigate the long-term impact of these protocols on patient outcomes. It should be emphasized that the correlations observed in this study do not establish causality. The associations between anticoagulation protocols and clinical outcomes should therefore be interpreted as exploratory rather than definitive evidence of causal effects. Larger randomized controlled trials are required to confirm whether simplified citrate anticoagulation directly improves these clinical parameters.

It is important to acknowledge the limitations of this study. The sample size was relatively small, and the study was conducted at a single center, which may limit the generalizability of the findings. Additionally, the study design was observational, and although efforts were made to control for confounding variables, the potential for unmeasured confounders cannot be completely ruled out. Future studies with larger, multicenter cohorts and prospective designs are warranted to corroborate the findings and provide a more comprehensive understanding of the impact of citrate anticoagulation protocols in HD. This study did not systematically measure RCA-specific parameters such as aPTT or post-HD ionized calcium due to protocol constraints. Future investigations incorporating these measurements could further elucidate the interplay between anticoagulation dynamics and inflammatory response modulation and cardiovascular outcomes in HD patients.

## 5. Conclusion

In conclusion, the simplified citrate anticoagulation protocol for HD demonstrates advantages across various domains including coagulation parameters, inflammatory markers, cardiovascular parameters, treatment response, and patient prognosis. The correlative analysis further emphasized the interconnectedness of anticoagulation protocols with various clinical and prognostic indicators. These findings have important implications for the individualized management of HD patients and underscore the need for further research to elucidate the mechanisms and long-term effects of different citrate anticoagulation protocols in this patient population.

## Author contributions

**Conceptualization:** Yunxia Wang, Jiajia Yang, Chun Wu.

**Data curation:** Yunxia Wang, Jiajia Yang, Chun Wu.

**Formal analysis:** Yunxia Wang, Jiajia Yang, Chun Wu.

**Funding acquisition:** Yunxia Wang, Chun Wu.

**Writing – original draft:** Chun Wu.

**Writing – review & editing:** Chun Wu.
